# IL-2 sensitivity and exogenous IL-2 concentration gradient tune the productive contact duration of CD8^+^ T cell-APC: a multiscale modeling study

**DOI:** 10.1186/s12918-016-0323-y

**Published:** 2016-08-17

**Authors:** Xuefeng Gao, Christophe Arpin, Jacqueline Marvel, Sotiris A. Prokopiou, Olivier Gandrillon, Fabien Crauste

**Affiliations:** 1 Inria team Dracula, Inria Antenne Lyon la Doua, Bâtiment CEI-2, 56 Boulevard Niels Bohr, 69603 Villeurbanne cedex, France; 2 Inserm, U1111, Lyon, F-69007 France; 3 CNRS, UMR5308, Lyon, F-69007 France; 4 Centre International de Recherche en Infectiologie, Université Lyon 1, Lyon, F-69007 France; 5 Ecole Normale Supérieure de Lyon, Lyon, F-69007 France; 6 Univ Lyon, ENS de Lyon, Univ Claude Bernard, CNRS UMR 5239, INSERM U1210, Laboratory of Biology and Modelling of the Cell, 46 allée d’Italie Site Jacques Monod, F-69007 Lyon, France; 7 Univ Lyon, Université Claude Bernard Lyon 1, CNRS UMR 5208, Institut Camille Jordan, 43 blvd. du 11 novembre 1918, F-69622 Villeurbanne cedex, France

**Keywords:** Multiscale modeling, CD8^+^ T cells, Differentiation pathway, IL-2, Activation threshold, Productive contact duration

## Abstract

**Background:**

The CD8^+^ T cell immune response fights acute infections by intracellular pathogens and, by generating an immune memory, enables immune responses against secondary infections. Activation of the CD8^+^ T cell immune response involves a succession of molecular events leading to modifications of CD8^+^ T cell population. To understand the endogenous and exogenous mechanisms controlling the activation of CD8^+^ T cells and to investigate the influence of early molecular events on the long-term cell population behavior, we developed a multiscale computational model. It integrates three levels of description: a Cellular Potts model describing the individual behavior of CD8^+^ T cells, a system of ordinary differential equations describing a decision-making molecular regulatory network at the intracellular level, and a partial differential equation describing the diffusion of IL-2 in the extracellular environment.

**Results:**

We first calibrated the model parameters based on in vivo data and showed the model’s ability to reproduce early dynamics of CD8^+^ T cells in murine lymph nodes after influenza infection, both at the cell population and intracellular levels. We then showed the model’s ability to reproduce the proliferative responses of CD5^hi^ and CD5^lo^ CD8^+^ T cells to exogenous IL-2 under a weak TCR stimulation. This stressed the role of short-lasting molecular events and the relevance of explicitly describing both intracellular and cellular scale dynamics. Our results suggest that the productive contact duration of CD8^+^ T cell-APC is influenced by the sensitivity of individual CD8^+^ T cells to the activation signal and by the IL-2 concentration in the extracellular environment.

**Conclusions:**

The multiscale nature of our model allows the reproduction and explanation of some acquired characteristics and functions of CD8^+^ T cells, and of their responses to multiple stimulation conditions, that would not be accessible in a classical description of cell population dynamics that would not consider intracellular dynamics.

**Electronic supplementary material:**

The online version of this article (doi:10.1186/s12918-016-0323-y) contains supplementary material, which is available to authorized users.

## Background

Biological systems comprise complex sets of biophysical, biochemical and cellular processes occurring at multiple temporal and spatial scales. The strong coupling of those scales, as well as the circular nature of causality in these systems [1], calls for the forging of new methods to explain emerging processes, notably by combining experimental data acquired at those different scales.

The immunological response appears like a promising test case in this respect because of its inherent complexity, and its large experimentalist community providing an ever-increasing level of experimental evidences, driving the modeling process.

Continuous models have been widely used to study immunological phenomena by simulating how average densities or concentrations of immune system agents (e.g., cells [2–5], or molecular species [6]) are changing over time and by identifying critical parameters that regulate the immune response. Although useful in simulating average responses of the immune system, it is difficult for continuous models to access the behavior of individual agents, in response to variations in parameters such as the duration of T cell and antigen-presenting cells (APC) interaction [7, 8], T cell cycling time [9] or intercellular interactions [10], that influence T cell responses.

Individual-based models are capable of coupling cell behaviors (e.g., migration, cell-cell interaction and division) with spatial concerns (e.g., diffusion of cytokines) and of yielding information on emerging population dynamics. For example, models have been developed to investigate the motility of T cells and cellular contact between T cells and APCs [11, 12], T cell strategy to search for antigens [13] or lymph node output efficiency [14, 15]. One important advantage of individual-based modeling approaches is that it allows incorporating quantitative characterization of the processes at the single-cell level. However, most of existing models neglect the intracellular mechanisms of cell decisions, hence limiting their explanatory power.

Baldazzi et al. [16] developed a hybrid model depicting lymphocyte recruitment and trafficking inside the lymph node, by coupling a stochastic agent-based description of cell interactions with a molecular diffusion described by partial differential equations (PDEs). Nevertheless, this model does not incorporate intercellular mechanisms controlling cells behavior but rather allows cells to sense and behave according to the concentration of specific chemokines in the surroundings. Another hybrid model was proposed by Santoni et al. [17] for describing T cell differentiation in the IgE-mediated hypersensitive reaction. At the intracellular level, this model comprises a genetic regulatory network (in Boolean formalism) for translating local cytokine signals (input) into explicit differentiation phenotype (output) through the activation of the represented genes (e.g., markers). At the cellular scale, the intracellular regulatory network is individually embedded in each CD4^+^ T cell using an agent-based model. The simulation result was able to reproduce cell population dynamics during a type I hypersensitivity reaction [17], however it could not generate realistic gene/protein expression profiles out of the intracellular network [18].

In the last decade, different models and tools [19–28] have been developed in an attempt to integrate processes across multiple scales and data from different resources (e.g., generated from experimental techniques) in order to explain immune responses. To further our understanding of the CD8^+^ T cell immune response, we have been modeling the CD8^+^ T cells behavior. We aimed at uncovering potential mechanisms and the impact of parameters that may help to explain some experimental observations, such as the responses of CD8^+^ T cells to different activation conditions. Since cell behavior, intracellular signaling and extracellular environment are inter-connected dynamical processes, an integration of these dynamics into a single platform requires multiscale modeling. Importantly, this type of models should produce quantitative simulation results that can recapitulate as well as explain a biological phenomenon at multiple scale levels [18].

In the present work, we aim at modeling how, in response to acute influenza virus infection, antigen-specific naïve CD8^+^ T cells become activated in the secondary lymphoid organs, rapidly proliferate and differentiate into effector lymphocytes that mediate viral clearance [7]. CD8^+^ T cells express T cell receptor (TCR) molecules on their surface that can recognize peptides derived from antigenic proteins bound to the products of the major histocompatibility complex (MHC) on APCs (mostly dendritic cells). When encountering cognate antigen-bearing APCs, CD8^+^ T cells reduce their rate of motility and eventually form prolonged contacts with APCs [29, 30], which are accompanied by the engagement of the TCR by peptide-MHC complexes. TCR engagement induces the synthesis of interleukin-2 (IL-2), which plays crucial roles in the growth and differentiation of CD8^+^ T cells [31]. Upon activation through signals delivered by the TCR and costimulatory molecules, T cells upregulate IL-2 receptor chains (IL-2R), i.e. IL-2-Rα (CD25), IL-2Rβ (CD122) and IL-2Rγ (CD132), and become highly sensitive to IL-2 [27]. Secreted IL-2 binds IL-2R (IL-2•IL-2R complex) to deliver a key signal for CD8^+^ T cell activation [31, 32]. In the presence of strong antigenic stimulation, CD8^+^ T cells are able to produce sufficient IL-2 for activation and proliferation [33].

In a different context, it has been shown that the proliferation of CD8^+^ T cells with a weak TCR stimulus requires IL-2 supplement, and T cell sensitivity to IL-2 strongly affects the extent of proliferation [34]. It is unknown how IL-2 sensitivity regulates CD8^+^ T cells behavior in the lymph node environment, where activated CD8^+^ T cells are influenced by the extra-cellular signals (e.g., cytokines and chemokines) as well as by interactions with APCs (duration, affinity/amount of antigenic peptides, co-stimulatory/inhibitory signals…) and other cell types, justifying the use of multiscale models for exploring these processes.

Finally, it has been observed that CD8^+^ T cells detach from APCs and undergo rapid proliferation after successful activation in vivo [35]. Some studies have demonstrated that the T cell-APC contact duration affects the quality and magnitude of an immune response [36] and memory T cell development [37]. Several studies have identified factors that influence T cell-APC contact duration including the maturation state of APCs [38], chemokines [39] and adhesion molecules (e.g., ICAM-1) [37]. Clearly, CD8^+^ T cell activation correlates with T cell-APC contact duration. However, it is still not clear how IL-2 influences T cell-APC contact duration.

We previously proposed a multiscale model [40] that qualitatively reproduces early CD8^+^ T cell immune responses in murine lymph nodes. At the intracellular level, the model comprises a set of ordinary differential equations (ODEs) describing the dynamics of a molecular regulatory network driving CD8^+^ T cell activation and differentiation. The intracellular processes are embedded in single CD8^+^ T cells and determine the cell phenotype through the dynamical functioning of the molecular regulatory network. At the cellular level, CD8^+^ T cells are described within a Cellular Potts model [41, 42]. Cells sense signals via surface receptor-ligand binding, involving either a direct cell-cell contact or diffusive molecules that are secreted by neighboring cells. Here, we use this model to address “*how and how much molecular inputs and the microenvironment can affect the behavior at the population level*.” [40].

We first calibrate the model parameters based on in vivo data. By mimicking acute infections, our model simulations quantitatively reproduce the CD8^+^ T cell population dynamics in murine lymph nodes as well as the dynamics of some key intracellular signals associated with CD8^+^ T cell activation and differentiation. In addition, using the same parameter values, our model proves to be able to reproduce another experimental situation for which it had not been designed: CD8^+^ T cell responses to weak antigenic stimulations with different concentrations of IL-2 supplement. The multiscale nature of the model allows to understand how different cellular responses might emerge from subtly different intracellular profiles, by linking the observation of transient short-term molecular events to long-term cell population dynamics. Finally, we show that CD8^+^ T cells-APC productive contact duration (i.e. the minimal T cell-APC contact time that allows a naïve T cell to become activated and to proliferate) is influenced by a balance between exogenous IL-2 availability and CD8^+^ T cell intrinsic IL-2 sensitivity, highlighting experimentally testable model-based predictions.

## Results and discussion

### Reproducing early CD8^+^ T cell immune response in murine lymph nodes

**Fig. 1:**
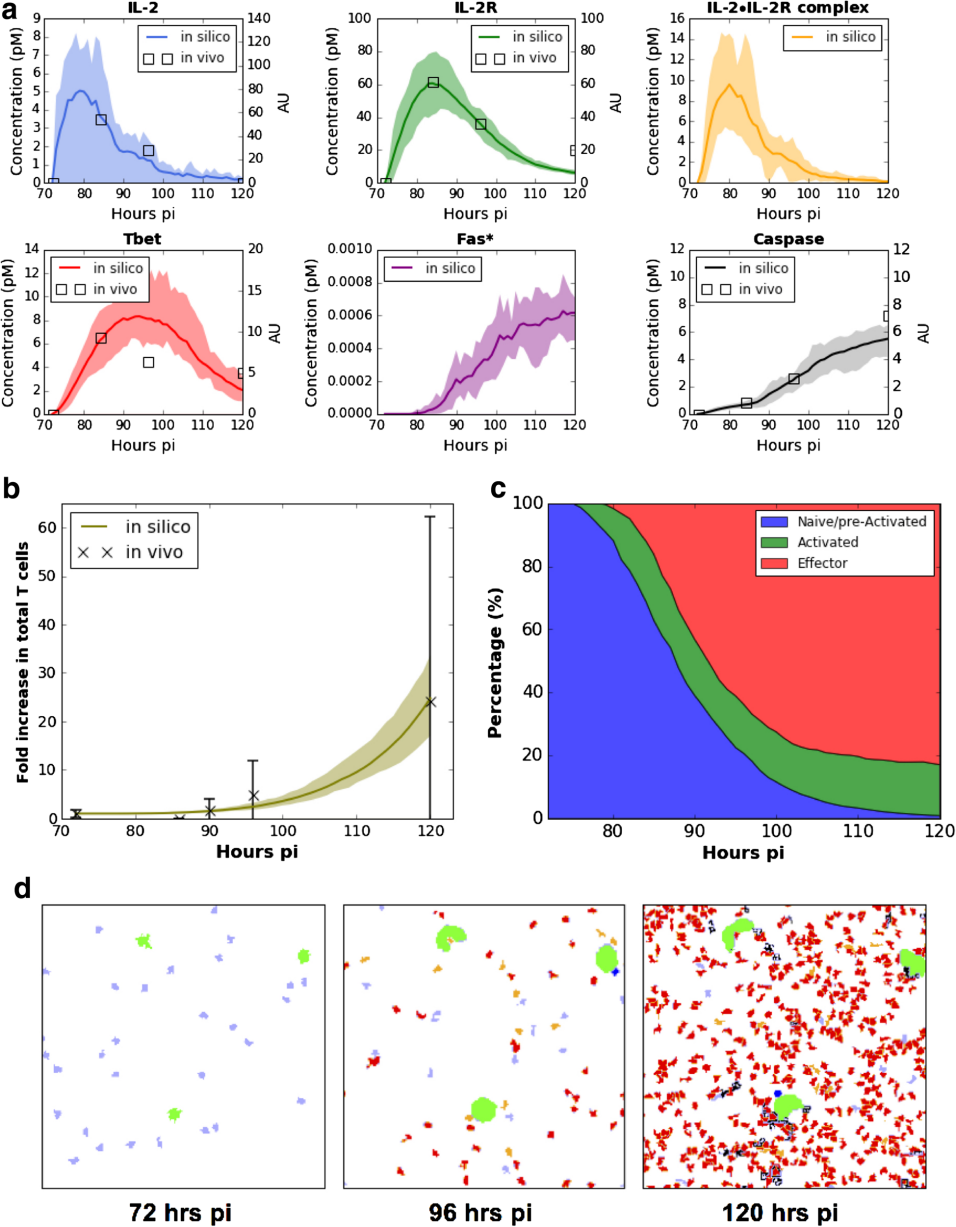
Reproduction of intracellular and cell dynamics data characterizing an early CD8+ T cell immune response. **a** Kinetics of IL-2, IL-2R, IL-2•IL-2R complex, T-bet, Fas* and cleaved Caspase from 72 h pi to 120 h pi. Molecular concentration is represented by squares (in vivo mRNA data, corresponding to the right-hand y-axis; *n* = 3; Caspase data are Caspase-3 data from ImmGen) or solid lines (simulation results, corresponding to the left-hand y-axis; mean value is indicated by a solid line, max and min values are upper and lower boundaries of the shaded areas respectively; *n* = 10 simulations). **b** CD8^+^ T cell dynamics, from 72 h pi to 120 h pi. Fold change of CD8^+^ T cell number is represented by cross marks for in vivo data (mean ± SD; cells were collected from 4 mice at each time point) and by a solid line for simulation results (mean value is indicated by a solid line, max and min values are upper and lower boundaries of the shaded area respectively; *n* = 10 simulations). **c** Proportion of the phenotypes within a simulated CD8^+^ T cell population (mean; *n* = 10 simulations). **d** Representative in silico simulation snapshots of 72 h, 96 h and 120 h pi. Color-coded are APCs (*green*), naïve cells (*light blue*), pre-activated cells (*dark blue*), activated cells (*orange*), effector cells (*red*) and apoptotic cells (*black*)

**Fig. 2:**
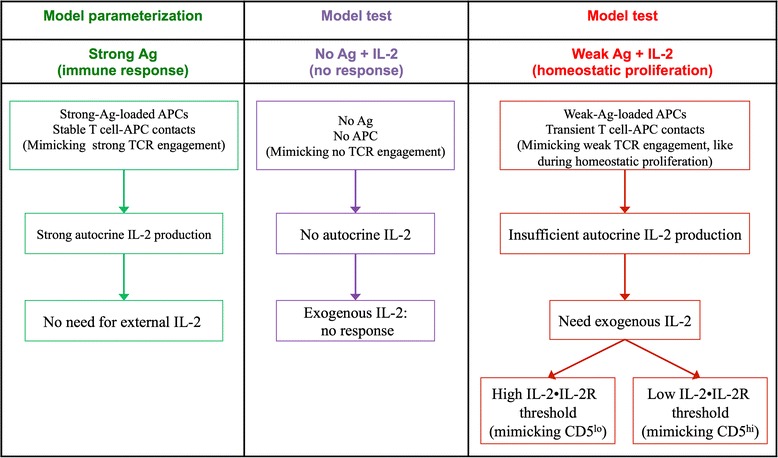
Schematic diagram of in silico experiments designed to test with our model CD8^+^ T cells productive responses to a weak TCR stimulation plus IL-2 supplement implemented in [30]

In order to refine the parameter values of our model, previously obtained in [40], we assessed its ability to reproduce two types of data generated at two different scales within 72–120 h post infection (pi) time frame:

1. Molecular data available through the *ImmGen* database (Fig. 1a) and
2. Cellular data consisting in the count of F5 transgenic cells in the lymph nodes of mice infected with Influenza virus (Fig. 1b).

Regarding the molecular behavior of our model, we observed that the average concentrations of IL-2 and IL-2R in the simulated cell population increase sharply in few hours post-infection (IL-2 and IL-2R curves in Fig. 1a). Around 78 h pi, a high level of IL-2•IL-2R (IL-2•IL-2R curve in Fig. 1a) appears in the simulations, which drives some pre-activated CD8^+^ T cells into the activation state (Fig. 1c). Following the emergence of pre-activated cells in the simulations, T-bet expression increases and peaks around 88 h pi at the population level (T-bet curve in Fig. 1a). With expansion of effector cells in the simulated populations, an increase in cellular contacts (effector-effector and effector-activated cells) elevates the frequency of Fas-FasL engagement which leads to an upregulation of Fas* (simulated Fas* curve in Fig. 1a), and the consequent cleavage/activation of Caspases (cleaved Caspase curve in Fig. 1a).

Regarding the cellular behavior of our model, the simulated CD8^+^ T cell population dynamics exhibits a pattern similar to the in vivo data (Fig. 1b), where cell proliferation starts at about 90 h pi, and then displays an exponential growth. Due to asymmetric partition of T-bet between daughter cells, effector phenotypes appear soon after the first T cell division (see simulation movie Additional file 1). Around 96 h pi, effector CD8^+^ T cells dominate the population in the simulations (Fig. 1c, d). Cell death appears sporadically following the emergence of effector cells and becomes frequent at later simulation points (see simulation movie Additional file 1; 120 h pi in Fig. 1d).

Overall, our model succeeds in reproducing the expected dynamics of CD8^+^ T cells in murine lymph nodes, at both the molecular and cellular scales. Importantly, it explains the cellular phenomena by generating in silico kinetics of the molecular species that match the in vivo data (from the *ImmGen* datasets). In addition, the model also makes some predictions such as the evolution of the proportion of the different cell types in a draining lymph node (Fig. 1c) or the evolution of the cleaved form of Caspase (Fig. 1a), as a function of time.

Parameter sensitivity (see Additional file 2) analyses indicate a robust performance of this model in reproducing the in vivo responses of CD8^+^ T cells to influenza virus infections. For example, small deviations of the T-bet or Caspase threshold values (e.g., ±1 pM of the control value, which corresponds to fits in Fig. 1a) do not significantly impair the simulation results (Additional file 2: Figure II and III). On the opposite, the IL-2•IL-2R threshold has a strong influence on the size of the total cell population and on the intracellular molecular dynamics (e.g., the levels of T-bet and cleaved Caspase) (Additional file 2: Figure I), indicating that the sensitivity of CD8^+^ T cells to IL-2 is an important factor regulating their responses.

### Exogenous IL-2 concentration and endogenous IL-2 sensitivity modulate the response of CD8^+^ T cells to weak TCR stimulation

The above simulation results illustrate 1) that with proper parameterization, our model is able to simulate the early phases of CD8^+^ T cell immune responses to strong antigens; 2) how the activation of naïve CD8^+^ T cells is induced by autocrine IL-2 production that results from strong TCR engagement; and 3) the process of effector cells development. We then wondered to which extent this model could reproduce other immunological conditions, for which it was not pre-parameterized. We decided to assess our model’s ability to reproduce part of the data reported by Cho et al. [34], which showed that with weak TCR signals induced by soluble CD3 monoclonal antibodies (mAb), IL-2 synthesis by CD8^+^ T cells is deficient and the presence of exogenous IL-2 is necessary for their activation and proliferation. In addition, the reactivity of CD8^+^ T cells to IL-2 has a good correlation with their CD5 expression, i.e., in vitro proliferative responses to IL-2 were stronger for CD5^hi^ cells than CD5^lo^ cells [34]. To further understand these phenomena at different scales as well as to test if our molecular network could mimic the in vitro IL-2-dependent proliferation of CD8^+^ T cells, we performed a set of in silico experiments (Fig. 2) based on the wet-lab experiments implemented in [34].

**Fig. 3:**
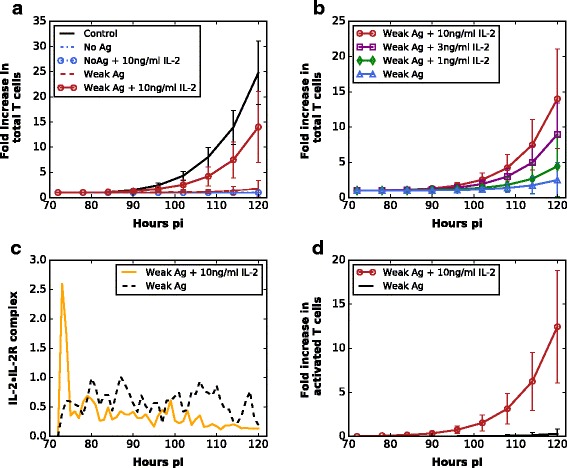
CD8^+^ T cell dynamics in various antigenic stimulation environments in silico. **a** Only the control population (stimulated with strong antigens) and the population submitted to a weak antigenic stimulation together with additional IL-2 supplement are able to proliferate (mean ± SD; *n* = 10 simulations). **b** CD8^+^ T cell dynamics in a weak antigenic condition with graded doses of IL-2 supplement (0.3–10 ng/ml) (mean ± SD; *n* = 10 simulations). **c** IL-2•IL-2R expression (mean; *n* = 10 simulations) and **d** the activated cells fold-increases (mean ± SD; *n* = 10 simulations) under the conditions of weak antigenic stimulation with or without 10 ng/ml IL-2

To simulate the response of CD8^+^ T cells to exogenous IL-2 under the antigen-free condition, we removed APCs from the computational domain and supplied naïve cells with IL-2 (middle column in Fig. 2). Unsurprisingly, at the population level neither cell activation nor proliferation is observed with or without IL-2 supplement (Fig. 3a; see simulation movies Additional files 3 and 4), which is in good agreement with the in vitro data [34]. At the intracellular level, no change in molecular expression is observed in the antigen-free simulations (Additional file 2: Figure IV), which explains the absence of cellular response.

To test the response of CD8^+^ T cells to a weak TCR stimulation, instead of providing the simulated cells with soluble CD3 mAb as described in [34], we loaded the APCs in our model with weak antigens aiming to mimic the low TCR signal strength (right column in Fig. 2). To this end, we simulated transient cellular contacts between naïve CD8^+^ T cells and weak-antigen-loaded APCs (right column in Fig. 2) based on the finding that stable T cell-APC interactions are antigen dependent [39]. This situation mimics in vivo homeostatic proliferation. We observe that with weak antigenic stimulation, proliferation of the CD8^+^ T cells is negligible in the absence of external IL-2 supplement (Fig. 3a; see simulation movie Additional file 5). Likewise, no cell proliferation is observed in the simulations supplemented with less than 0.3 ng/ml IL-2 (Fig. 3b). This is in agreement with observations that proliferative responses of naïve CD8^+^ T cells in the context of weak TCR ligation depend on IL-2, both in vitro [34] and in vivo. Up to that stage, our model is then able to reproduce non-straightforward in vitro biological observations for which it had not been parameterized.

By contrast, a relative stronger proliferative response occurs when supplying the simulation domain with 3 ng/ml IL-2 or higher (Fig. 3b; see simulation movie Additional file 6), as highlighted in vitro by Cho et al. [34]. By comparing the intracellular dynamics of CD8^+^ T cells stimulated by weak antigens with and without IL-2 supplement (Additional file 2: Figure IV), a transient high level of IL-2•IL-2R complex arising from IL-2 addition is captured at early simulation time points (Fig. 3c). In fact, this transient peak of IL-2•IL-2R is sufficient to drive some naïve CD8^+^ T cells into the activation state (Fig. 3d) and then leads to a subsequent activation and proliferation of CD8^+^ T cells, observable several hours later. Given the fast decay rate of IL-2 in vivo (average serum half-life is 3.7 min [43]), successful activation with weak TCR stimulation requires that naïve CD8^+^ T cells encounter APCs at early time points so that they can up-regulate IL-2R before the IL-2 concentration in the surrounding environment becomes insufficient. Hence, our modeling approach highlights molecular mechanisms explaining why exogenous IL-2 is the key for the proliferative responses of CD8^+^ T cells to a low TCR signal induced by weak antigens in vivo. It thereby explains the in vitro CD8^+^ T cell proliferation induced by a weak TCR stimulation with IL-2 supplement, thus reproducing and explaining biological observations it had not been designed to describe.

**Fig. 4:**
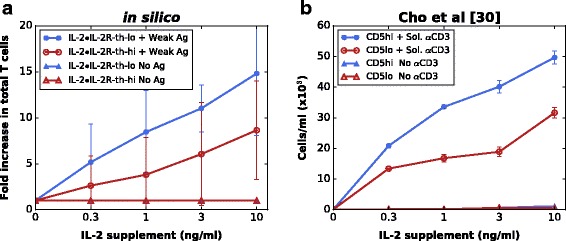
Sensitivity of CD8^+^ T cells to IL-2 correlates with the strength of proliferation response in the context of weak TCR stimulation. **a** Simulation results: proliferative responses of IL-2•IL-2R-threshold-low and -high CD8^+^ T cells to graded doses of IL-2 supplement (0.3–10 ng/ml) with a weak antigenic stimulation (Weak Ag) or in antigen-free conditions (No Ag) (mean ± SD; *n* = 10 simulations). Total CD8^+^ T cell counts are measured at 120 h pi. **b** In vitro results (data come from Fig. 5a in [30]): in vitro proliferation of B6 naïve CD5^lo^ and CD5^hi^ CD8^+^ T cells to a low concentration of soluble CD3 mAb (0.1 μg/ml) with or without graded concentrations of IL-2 supplement (0.3–10 ng/ml)

Naïve CD8^+^ T cells display different IL-2 sensitivities in vitro; this is correlated with CD5 expression: CD5^hi^ CD8^+^ T cells are highly sensitive to IL-2 compared to CD5^lo^ CD8^+^ T cells [34]. To further examine the role of IL-2 signaling in controlling CD8^+^ T cells response to weak antigens, we modeled the responses of CD5^lo^ cells and CD5^hi^ cells by setting the required threshold for IL-2•IL-2R-induced cell activation at different levels (high IL-2•IL-2R-threshold (55 pM) for CD5^lo^ cells and low IL-2•IL-2R-threshold (47 pM) for CD5^hi^ cells) (right-hand column in Fig. 2). Simulation results show that cells with a low IL-2•IL-2R-threshold give rise to larger populations than cells with a high IL-2•IL-2R-threshold (Fig. 4a). Qualitatively, these simulation results are in excellent agreement with in vitro simulated homeostatic proliferations shown in [34] (Fig. 4b).

Even though CD5 levels of CD8^+^ T cells have been shown to be correlated with their responsiveness to IL-2 stimulations in weak antigenic contexts (low-level TCR signals arising from neighboring T cells with self-MHC-I ligands or weak-antigen-loaded APCs inducing transient contacts), our simulation results additionally suggest that the proliferative response of CD8^+^ T cells to low-level TCR signals is regulated by a fine tuning of their dynamical response to IL-2 and the IL-2 concentration in the extracellular environment. From the single cell level, our simulations indicate a balance between the IL-2•IL-2R threshold level and the IL-2•IL-2R formation efficiency determining the activation of individual CD8^+^ T cells, which is further detailed in the following section. In conclusion, our model proved to be able to reproduce CD8^+^ T cell responses to multiple activation conditions (e.g., in vivo response to antigens and homeostatic proliferation) and to make experimentally testable predictions (that are further developed in the following section).

### Exogenous IL-2 concentration and endogenous IL-2 sensitivity tune the productive contact duration of CD8^+^T cell-APC

**Fig. 5:**
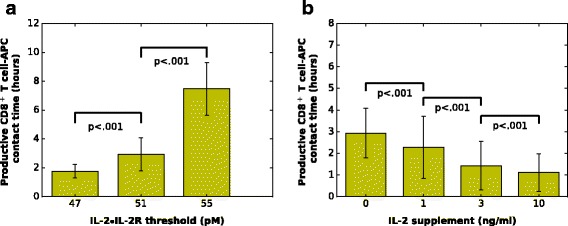
The productive CD8+ T cell-APC contact time varies with **a** the IL-2•IL-2R threshold level of individual T cells (without IL-2 supplement; mean ± SD; *n* = 10 simulations) and **b** the exogenous IL-2 concentration (with IL-2•IL-2R threshold = 51 molars; mean ± SD; *n* = 10 simulations)

Next we measured the impact of different IL-2•IL-2R threshold levels on the productive T cells-APC contact duration in response to a strong antigenic stimulation. We simulated the responses of IL-2•IL-2R-threshold-high and IL-2•IL-2R-threshold-low CD8^+^ T cells to strong antigens, and measured their productive contact duration with APCs (the minimal contact time between a naïve T cell and an APC that gives rise to a proliferative T cell). Simulations show that IL-2•IL-2R-threshold-low cells have significantly reduced TCR-APC engagement duration (contact time < 2 h) than IL-2•IL-2R-threshold-high cells (contact time > 7 h) (Fig. 5a). This result suggests that IL-2 sensitivity of individual naïve CD8^+^ T cells may influence their requirement for a prolonged contact with APCs in vivo. Accordingly, our model predicts that CD5^hi^ CD8^+^ T cells may have shorter productive contact time with APCs comparing to their CD5^lo^ counterparts in vivo.

In addition to the IL-2 sensitivity, the IL-2•IL-2R formation rate also affects the productive contact duration of CD8^+^ T cell-APC, which partially depends on the amount of IL-2 in the extracellular environment during TCR engagement. Indeed, we observed that a high concentration of exogenous IL-2 (e.g., 10 ng/ml) induced fast formation of IL-2•IL-2R in CD8^+^ T cells, which consequently shortened the productive contact time of T cell-APC (Fig. 5b). Hence, the concentration of exogenous IL-2 could affect CD8^+^ T cell activation by decreasing the T cell-APC contact time necessary for productive activation. This result is in accordance with a study by Filby et al. [44] showing that Fyn^−/−^ CD8^+^ T cells producing IL-2 more efficiently require a shorter TCR engagement duration. Thus this experimental evidence supports our prediction that T cells require shorter contacts with APCs if they can access abundant IL-2 by autocrine production or from the neighboring IL-2 producing cells (e.g., CD4^+^ T cells).

Taken together, the productive contact time of T cell-APC is thus influenced by both the intrinsic IL-2 sensitivity of individual CD8^+^ T cells and the IL-2 level in the extracellular environment, a prediction that could be tested experimentally, at least partially, by modifying the available IL-2 quantities in the extracellular environment at different times during the early events of the response and measuring the subsequent expansion of the population. In addition, our model indicates that CD5^hi^ cells require shorter contact times with APCs to become activated, which could help them entering a proliferative state faster than their CD5^lo^ counterparts.

## Conclusions

In this paper, we used a multiscale model of CD8^+^ T cell dynamics, based on explicit descriptions of cellular dynamics with a Cellular Potts model and with cellular decision-making at the single cell level controlled by a molecular regulatory network. The main objective was to address the following questions: how does IL-2 supplement combined with a weak antigenic stimulation induce a visible response? How does heterogeneity within the naïve CD8^+^ T-cell pool (e.g., different sensitivity to the stimulations) may explain variations in the T cell responses? Answers to those questions are not straightforward due to the dynamical, multiscale nature of the CD8^+^ T cell behavior. We showed that, by coupling the different relevant scales, our modeling approach shed new light on those questions.

After parameter calibration based on in vivo data, this model was capable of recapitulating the population and intracellular molecular dynamics of CD8^+^ T cells in murine lymph nodes following an influenza virus infection in a strong antigenic-stimulation context. Overall, the simulation results are in good agreement with in vivo experimental data at multiple scales, and showed a robust performance in reproducing the response of CD8^+^ T cells to strong antigenic stimulations.

In order to assess the robustness of the model (with parameter values in Additional file 2: Tables II, III and IV, calibrated based on the data in Fig. 1), we decided to assess its ability to simulate other physiological conditions for which it had not been parameterized for, e.g. in vivo homeostatic proliferation, as modeled in vitro by Cho et al. [34]. We thus studied the responses of CD8^+^ T cells to IL-2 supplement in an antigen-free condition or under a weak antigenic stimulation (weak-antigen-loaded APCs). No immune response was observed in an antigen-free condition with or without IL-2 addition. With a weak TCR signal provided by contacting with weak-antigen-loaded APCs, addition of large concentrations of exogenous IL-2 was required for CD8^+^ T cells to overcome the activation threshold. Compared to CD8^+^ T cells stimulated with a weak TCR stimulation alone, exogenous IL-2 led to a transient high level of IL-2•IL-2R complex at early time points (Fig. 3c) which was identified as a key force driving some naïve CD8^+^ T cells into the activated state (Fig. 3d). A simple examination of the cellular (Fig. 3a) or molecular (Fig. 3c) levels separately would not have yielded such kind of insight as the multiscale integration permitted. It is indeed practically difficult to predict, without a multiscale model, how a seemingly tenuous difference (as seen in IL-2•IL-2R kinetics in the presence or absence of IL-2 at early time points (Fig. 3c)) can translate into a none-or-all type of response at the cellular level.

Furthermore, by manipulating the IL-2•IL-2R threshold level of single CD8^+^ T cells and implementing CD5^hi^ and CD5^lo^-like CD8^+^ T cells, the model allowed a reproduction of responses involving high affinity (CD5^hi^) cells, for which it had been developed, and low affinity (CD5^lo^) cells, for which it had not been designed. This stressed the model robustness to high affinity and low affinity clones. Second, our simulations of the proliferative responses of CD5^hi^ and CD5^lo^ CD8^+^ T cells to a weak antigenic stimulation in the presence of different concentrations of IL-2 supplement is in good accordance with in vitro experimental data [34]. This result suggests that the difference between CD5^hi^ and CD5^lo^ cells is partially determined by their endogenous sensitivity to IL-2.

It has been shown that stop signals transmitted by antigen-bearing APCs towards T cells are crucial for T cell activation, since such signals enhance long-lived interactions between T cells and APCs [12]. However, this hypothesis has been challenged by naïve T cells becoming activated via brief interactions (<10 min) with antigen-bearing dendritic cells when placed in a collagen matrix [45, 46]. In our study, a reduced productive contact time of T cell-APC was observed when lowering the IL-2•IL-2R threshold for activation, suggesting that the sensitivity of CD8^+^ T cells to IL-2 might affect their TCR engagement requirements. Some studies demonstrate that the history of T cell-APC interactions experienced by individual T cells may contribute to the heterogeneity of T cell responses [47]. Other studies suggest the sensitivity of individual T cells to the activation signals may predetermine the phenotypes of CD8^+^ T cells [7, 48]. Taken together, these results indicate that heterogeneity within the naïve CD8^+^ T-cell pool (e.g., cells have different sensitivity to the stimulations) may partially explain variations in the T cell responses to antigens, including T cell-APC contact duration, fate decisions and phenotype development.

Although we tried to incorporate as much relevant biological information as we thought was strictly necessary for reproducing the data we had, there is nevertheless ample room for improving our model. For example, comparing to morphology of dendritic cells in vivo (with dendrites extended from their center mass), the surface area of our 2D simulated APCs is small, which limits their contact frequency with T cells. This limitation is in part the reason that upper range of our simulation population size is below the observed data (Fig. 1b).

The use of 2D model instead of a 3D model can also be seen as a limitation. The computational cost of a 3D multiscale model is higher than the one of a 2D model, and mainly justifies our choice to use a 2D model (a 3D model could however be easily parameterized to reproduce experimental data and investigate CD8^+^ T cell properties, provided that a large amount of simulations can be performed in a reasonable amount of time). Nevertheless, the result on the predicted productive contact duration between T cells and APCs in our model does not a priori depend on a 2D or a 3D model, since it mainly relies on the intracellular dynamics and the intracellular molecular model is deterministic in our current model.

In addition, a refined molecular network incorporating the Eomesodermin (Eomes) transcription factor could allow the generation of memory cells, based on some studies showing that Eomes upregulation together with T-bet downregulation induces the formation of memory T cells [49]. Extracellular regulating actors, like CD4^+^ T cells that can, among other things, contribute to IL-2 secretion [50], and environmental variables like inflammation or aging could be considered, leading to refinements of the model. One of the main limitations here would be the lack of in vivo lymph node dynamical data that would be critically needed for proper parameterization and validation of the added model elements. For example, a time-dependent characterization in the evolution of the different cellular subpopulations would permit to assess the validity of our predictions (Fig. 1c). Furthermore, it would be highly valuable to obtain single-cell values of mRNA expressions for the molecular actors of our network, and their time-dependent evolution during the response. The recent advent of single-cell transcriptomics (see e.g. [51]) could be helpful in that respect.

In conclusion, our multiscale model study indicates that the CD8^+^ T cell differentiation process is an acquired property resulting from dynamical interactions between the intracellular molecular network and environmental factors. Therefore, it is an ideal platform for integrating data obtained at different scales and studying how their coupling explains cellular behaviors of T cells.

## Methods

### Experimental procedures

Two hundred thousands CD8^+^ T cells from CD45.1^+^ F5 TCR transgenic (tg) mice (B6.SJL-Ptprc^a^Pepc^b^/BoyCrl-Tg(CD2-TcraF5, CD2-TcrbF5)1Kio/Jmar) recognizing the NP68 epitope were transferred by retro-orbital injection in 6–8 week old congenic CD45.2^+^ C57BL/6 mice (C57BL6/J). The day after recipient mice were inoculated intranasally with 2.10^5^ TCID_50_ of an H1N1 Influenza virus expressing the NP68 epitope. From Day 3 to 5.5 post-infection, the lymph node draining the site of infection (lung, mediastinal lymph node) was harvested and the number of F5 transgenic CD8 responder T cells was assessed by flow cytometry, based on CD8/CD45.1/CD45.2 expression, to distinguish F5 TCR-tg responder (CD45.1^+^CD45.2^−^) from host (CD45.1^−^CD45.2^+^) CD8+ T cells.

### mRNA profile

Subcellular mRNA data comes from the *ImmGen* project, http://www.immgen.org. According to the information provided on ImmGen.org, the in vivo mRNA data (presented in Fig. 1a) were generated from spleen OT-1 CD8^+^ T cells during the course of a response to Listeria-OVA infection, and normalized using the robust multi-array algorithm (so normalized values are between 10 and 20,000). We converted normalized data into fold change for performing model validation, based on the expression level in naïve CD8^+^ T cells $\left( fold\  change=\frac{given\  data}{expression\  in\  naive\  cell}-1\right)$.

It must be noted that no in vivo mRNA data is available for the signaling quaternary IL-2•IL-2R complex, which is used in this model (see section Modeling below). In addition, in vivo mRNA data for Fas, which correspond to the sum of inactivated and activated forms of Fas, show an almost constant level of expression throughout the response (data not shown). This information has been used to describe the dynamics of Fas and its activated form in the model (we assume a constant level of expression of the total quantity of Fas, and describe only the dynamics of the activated form, Fas*), consequently there is no data available for the dynamics of Fas*.

### Modeling

For the reader’s convenience, we present in this section our multiscale model, previously introduced in [40]. It consists in the coupling of molecular and cellular dynamics of CD8^+^ T cells through an explicit description of single cell dynamics (movements, interactions, cycling) within the whole population (cellular scale) and a molecular regulatory network-based decision-making at the single cell level (subcellular scale). In addition, diffusion of interleukin-2 (IL-2) in the extracellular environment is explicitly described and contributes to cell communication. A detailed description of the model construction and parameterization can be found in Additional file 2.

#### Cellular scale: a Cellular Potts model

**Fig. 6:**
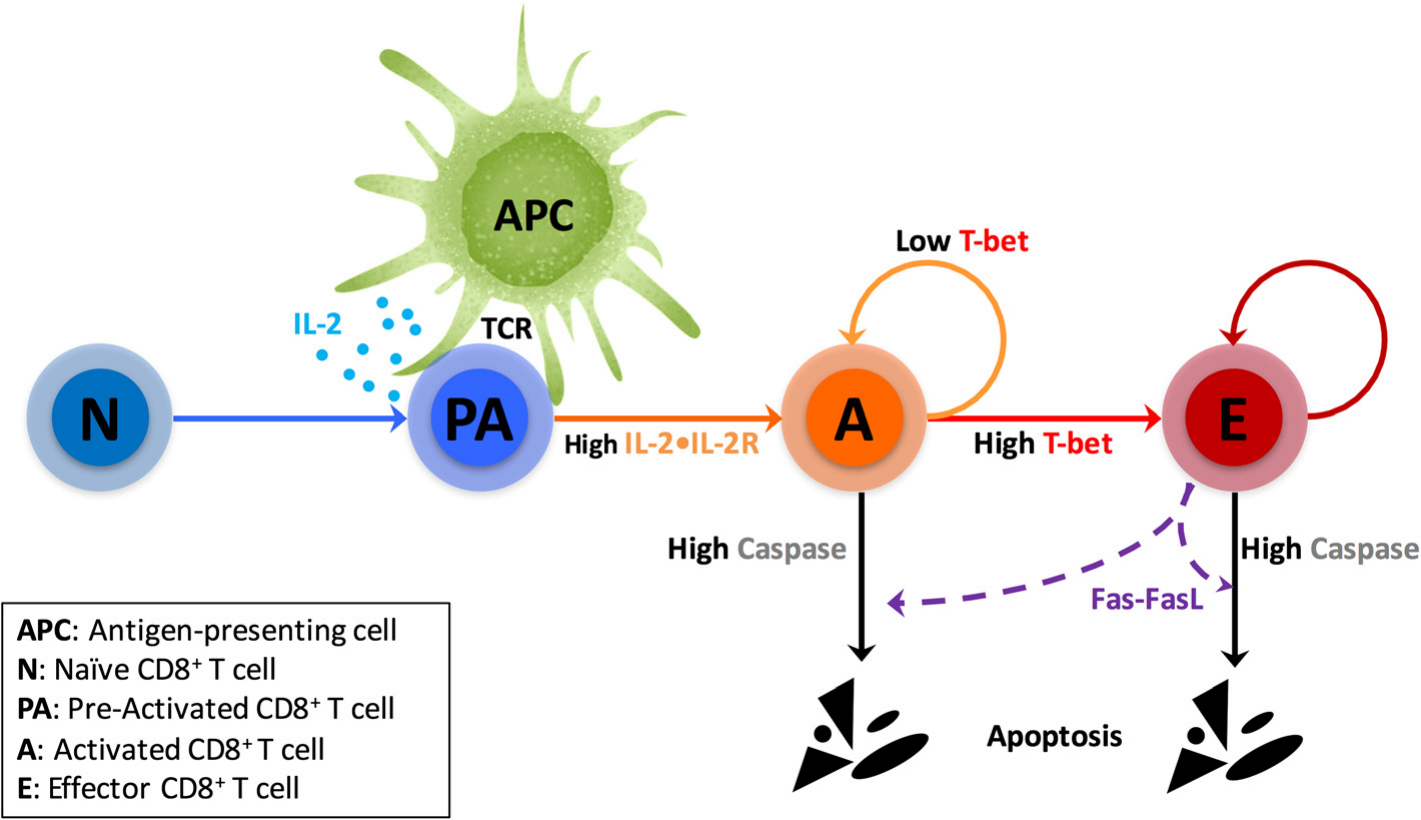
Schematic diagram of the linear in silico activation pathway of CD8^+^ T cells. A naïve CD8^+^ T cell becomes pre-activated when it contacts an APC. The pre-activated T cell evolves to an activated cell when its IL-2•IL-2R complex exceeds a threshold. The activated T cell breaks contact with APC and enters cell cycle. At the end of an activated T cell’s division, the daughter cells can differentiate into effector phenotype if their T-bet level exceeds a threshold. Effector T cells keep proliferating leading to the population expansion. The apoptosis of activated and effector T cells involves the generation of cleaved Caspases (above a threshold), which are induced by Fas-FasL signaling

At cellular scale, we used a Cellular Potts model to describe cellular behaviors. The implementation of the Cellular Potts model, including the choice of initial conditions and the characterization of each cell type (CD8^+^ T cells and antigen-presenting cells), is detailed in Additional file 2. We assumed a linear differentiation pathway for CD8^+^ T cells (Fig. 6), consisting in naïve T cells differentiating in pre-activated cells, then in activated cells, and finally in effector cells, provided cells did not die while cycling. The state of a dynamical molecular regulatory network (next section) determines individual CD8^+^ T cell’s phenotype and fate, as follows.

In the simulations, naïve CD8^+^ T cells move constitutively and randomly until encountering APCs (see simulation movie Additional file 1). When a naïve CD8^+^ T cell encounters an APC, it becomes pre-activated upon TCR engagement. During their pre-activated state, T cells maintain a stable attachment with APCs, and secrete IL-2 to the surrounding environment (see simulation movie Additional file 1). The secreted IL-2 combines with IL-2 receptors (IL-2R) to form the signaling quaternary IL-2•IL-2R complex on the cell membranes. Studies indicate that CD8^+^ T cell proliferation ensues when reaching a certain activation condition (e.g., an activation signal exceeding a given threshold) [7]. Moreover, in vitro studies showed that IL-2 supplement can synergize with weak TCR ligation to induce the proliferation of naïve CD8^+^ T cells [34, 52], underlining the crucial role of IL-2 signaling in CD8^+^ T cell activation. Hence, we define an IL-2R occupancy level as the activation threshold. When the IL-2•IL-2R level within a pre-activated CD8^+^ T cell overcomes this threshold, the T cell becomes activated, detaches from APC and enters the cell cycle.

It has been shown that T-bet over-expression promotes the formation of effector phenotype [53]. Hence, we assume that in our model a cell differentiates into an effector cell when its T-bet level overcomes a predefined threshold value (Fig. 6).

We determine CD8^+^ T cell death by setting a threshold of cleaved Caspase, given the broadly known role of caspases in immune cell apoptosis [54]. Following differentiation of naïve CD8^+^ T cells into effector phenotype, Fas-ligand (FasL) is expressed on cell surface [55]. Fas is expressed on the activated/effector CD8^+^ T cell surface which can bind FasL on the surface of a contacting T cell [55]. Cellular contacts between Fas-expressing cells and FasL result in cleaved Caspase activation and apoptosis [56, 57].

#### Subcellular scale: a system of ODEs describes the molecular regulatory network of single CD8^+^ T cells

**Fig. 7:**
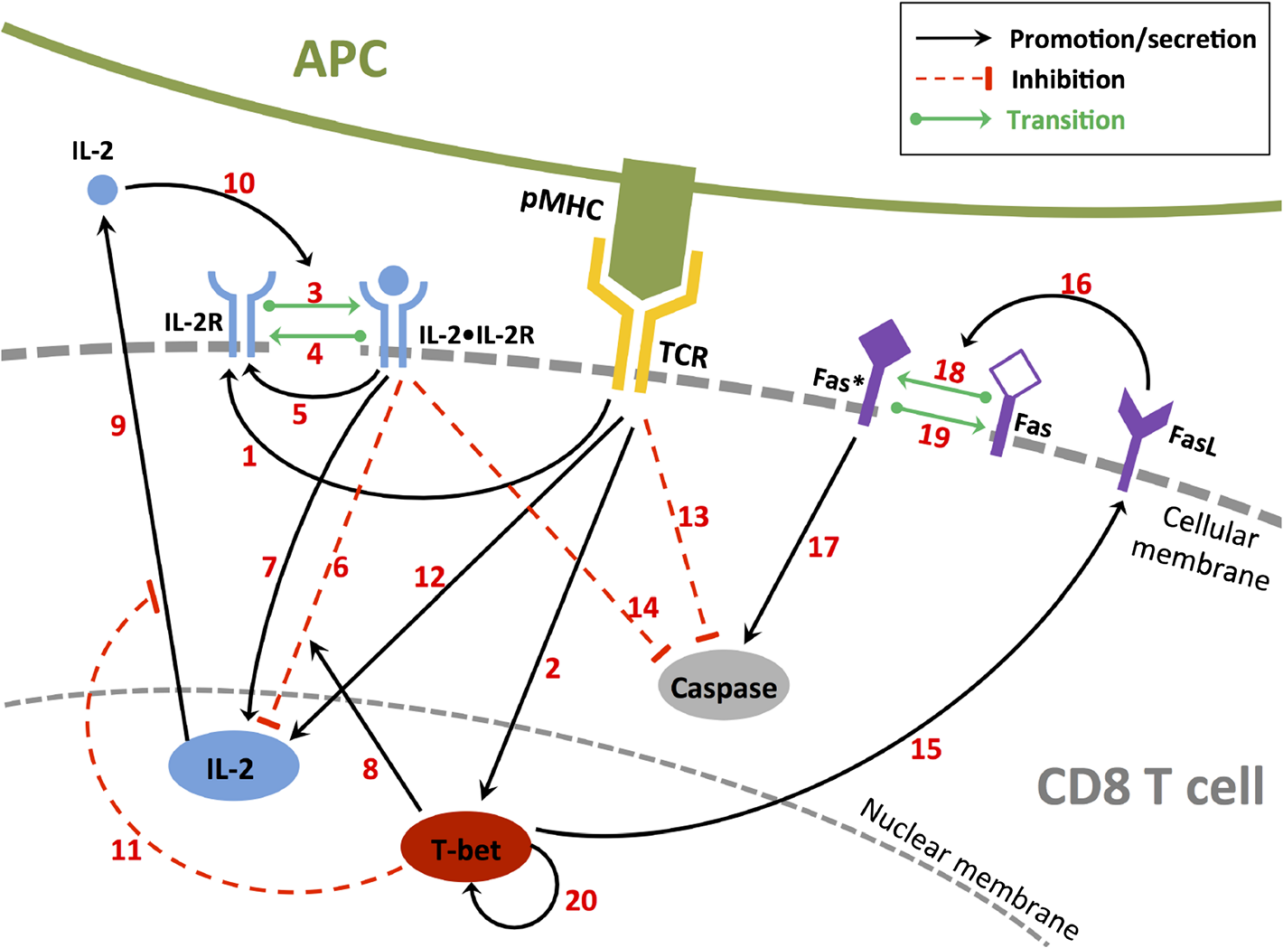
A simplified molecular regulatory network of a CD8^+^ T cell

Previously, we proposed a simplified molecular regulatory network [40] depicting some crucial signaling (Fig. 7) involved in the early CD8^+^ T cell immune response. The dynamical state of the molecular regulatory network controls the CD8^+^ T cell’s phenotype and fate (see previous section). The description of this molecular signaling pathway is summarized in Fig. 7 and follows:

- Arrow 1: TCR activation induces IL-2R expression [6, 31].
- Arrow 2: TCR activation induces T-bet expression [49].
- Arrow 3: IL-2 binds to IL-2R to form the IL-2•IL-2R complex [6, 31, 32, 58].
- Arrow 4: Disassociation of IL-2•IL-2R recycles the IL-2R to T cell surface [32].
- Arrow 5: IL-2•IL-2R enhances the expression of non-activated IL-2R [32].
- Arrow 6: IL-2•IL-2R inhibits IL-2 gene expression [31, 32, 59, 60].
- Arrow 7: IL-2•IL-2R enhances IL-2 gene expression [31, 32, 59].
- Arrow 8: T-bet promotes IL-2 gene suppression via enhancing the IL-2R inhibition of IL-2 gene [59, 60].
- Arrow 9: IL-2 molecule secretion [6, 31, 32, 59].
- Arrow 10: IL-2 enhances IL-2•IL-2R formation [6, 31, 32, 59].
- Arrow 11: T-bet inhibits IL-2 secretion [60, 61].
- Arrow 12: TCR activation induces IL-2 gene expression [6, 31, 32].
- Arrow 13: TCR activation inhibits Caspase cleavage/activation [57, 63].
- Arrow 14: IL-2•IL-2R inhibits Caspase cleavage/activation [62].
- Arrow 15: T-bet induces FasL expression on effector cell’s surface [59, 64].
- Arrow 16: FasL induces activation of Fas via cell-cell contact [59].
- Arrow 17: Activated Fas (Fas*) induces Caspase cleavage/activation [57].
- Arrow 18: Activation of Fas [59].
- Arrow 19: Deactivation of Fas* [57].
- Arrow 20: T-bet expression is self-enhanced (i.e., a positive feedback loop) [65, 66].

One may note that following TCR engagement an autocrine loop is activated, involving IL-2 and its receptor IL-2R (arrows 7, 9 and 10). However, this positive loop is negatively regulated [31, 32, 59, 60] on a longer time scale (arrow 6) via different signaling pathways involving Blimp-1 (not explicitly described in the model) and enhanced by T-bet (arrow 8), hence controlling IL-2 production throughout the entire response. The dynamics of the regulatory network (Fig. 7) is converted into the following nonlinear ODE model:

1 $$ \boldsymbol{I}\boldsymbol{L}\boldsymbol{\hbox{-}}\boldsymbol{2}\boldsymbol{R}:\kern1em \frac{d\left[R\right]}{dt}={\lambda}_{R1}f(APC)+\left({\mu}_{IL2}^{-}+{\lambda}_{R2}\right)\left[L\bullet R\right]-{\mu}_{IL2}^{+}\left[IL{2}^{cm}\right]\left[R\right]-{k}_R\left[R\right] $$

2 $$ \begin{array}{l}\boldsymbol{I}\boldsymbol{L}\boldsymbol{\hbox{-}}\boldsymbol{2}\bullet \boldsymbol{I}\boldsymbol{L}\boldsymbol{\hbox{-}}\boldsymbol{2}\boldsymbol{R}:\kern1em \frac{d\left[L\bullet R\right]}{dt}={\mu}_{IL2}^{+}\left[IL{2}^{cm}\right]\left[R\right]-{\mu}_{IL2}^{-}\left[L\bullet R\right]-{k}_e\left[L\bullet R\right]\\ {}\end{array} $$

3 $$ \boldsymbol{T}\boldsymbol{\hbox{-}}\boldsymbol{bet}:\kern1em \frac{d\left[ Tb\right]}{dt}={\lambda}_{T1}f(APC)+{\lambda}_{T2}\frac{\left[ Tb\right]}{\lambda_{T3}+\left[ Tb\right]}\left[ Tb\right]-{k}_T\left[ Tb\right] $$

4 $$ Activated\;\boldsymbol{F}\boldsymbol{a}\boldsymbol{s}:\kern1em \frac{d\left[Fs\right]}{dt}=H{\mu}_F^{+}\left[T{b}^{cm}\right]\left(\frac{\lambda_F}{k_F}-\left[Fs\right]\right)-{\mu}_F^{-}\left[Fs\right]-{k}_F\left[Fs\right] $$

5 $$ Cleaved\;\boldsymbol{Caspase}:\kern1em \frac{d\left[Cas\right]}{dt}={\lambda}_{C1}\left(\frac{1}{1+{\lambda}_{C2}\left[L\bullet R\right]}\right)\cdot \left(\frac{f(APC)}{1+{\lambda}_{C3}f(APC)}\right)+{\lambda}_{C4}\left[Fs\right]-{k}_C\left[Cas\right] $$

where [*R*], [*L* • *R*], [*Tb*], [*Fs*] and [*Cas*] are the respective concentrations in IL-2 receptors, activated form of the IL-2 receptors, Tbet, activated Fas, and cleaved caspases. Parameters *λ* denote strengths of feedback controls, parameters *k* are decay rates of the corresponding subscripted variables, *μ*^+^ and *μ*^−^ are association and dissociation rates for IL2-R and Fas. The term *f* (*APC*) in equations (1), (3) and (5) represents the strength of TCR signaling, assumed here to be equal to the number of contacting APCs (and equal to zero when a CD8^+^ T cell is not contacting an APC). Two additional terms, [*IL2*^*cm*^] and [*Tb*^*cm*^], account for interactions with the external environment and neighbor CD8^+^ T cells: [*IL2*^*cm*^] represents the concentration of IL-2 at the boundary of the CD8^+^ T cell, in the extracellular environment, while [*Tb*^*cm*^] represents the sum of all T-bet concentrations from contacting surrounding CD8^+^ T cells and implicitly describes Fas-ligand induced caspase activation. Parameters values are described in Additional file 2: Table II.

#### Extracellular scale: a PDE describes IL-2 production and diffusion in the extracellular environment

Taking interactive dynamics (arrow 6–9, 11 and 12 in Fig. 7) into account, we describe IL-2 dynamics in the extracellular environment (mainly diffusion and degradation) by using a partial differential equation (PDE), as follows:

6 $$ \frac{\partial \left[IL2\right]}{\partial t}=D{\nabla}^2\left[IL2\right]+\left({\lambda}_{R3}\frac{\left[L\bullet R\right]}{\lambda_{R4}+\left[L\bullet R\right]}+{\lambda}_1f(APC)\right)\frac{1}{1+{\lambda}_4\left[ Tb\right]}-\delta \left[IL2\right] $$

The parameter *D* is the diffusion coefficient of extracellular IL-2, *δ* its decay rate, and parameters *λ* represent strengths of various feedback controls. All parameters values are described in Additional file 2: Table IV.

## Abbreviations

APC, antigen-presenting cell; FasL, Fas ligand; IL-2, interleukin-2; IL-2R, interleukin-2 receptor; mAb, monoclonal antibody; MHC, major histocompatibility complex; ODE, ordinary differential equation; PDE, partial differential equation; TCR, T-cell receptor; tg, transgenic

## Additional files

Additional file 1: CompuCell3D simulation of CD8^+^ T cells activation with strong antigenic stimulation. This movie is related to Fig. 1 in the main text and Additional file 2: Figure IVA. For all the simulation movies, the color-coded are APCs (green), naïve cells (light blue), pre-activated cells (dark blue), activated cells (orange), effector cells (red) and apoptotic cells (black). The diffusion field shows the IL-2 concentration in molars. (MOV 8752 kb) Additional file 2: This file contains the model implementation and parameterization. (DOCX 523 kb) Additional file 3: CompuCell3D simulation of CD8^+^ T cells under antigen-free condition. This movie is related to Fig. 3 in the main text and Additional file 2: Figure IVB. The color-coded are APCs (green), naïve cells (light blue), pre-activated cells (dark blue), activated cells (orange), effector cells (red) and apoptotic cells (black). The diffusion field shows the IL-2 concentration in molars. (MOV 1368 kb) Additional file 4: CompuCell3D simulation of CD8^+^ T cells activation with 10 ng/ml IL-2 supplement under antigen-free condition. This movie is related to Fig. 3 in the main text and Additional file 2: Figure IVC. The color-coded are APCs (green), naïve cells (light blue), pre-activated cells (dark blue), activated cells (orange), effector cells (red) and apoptotic cells (black). The diffusion field shows the IL-2 concentration in molars. (MOV 1445 kb) Additional file 5: CompuCell3D simulation of CD8^+^ T cells activation with weak antigenic stimulation. This movie is related to Fig. 3 in the main text and Additional file 2: Figure IVD. The color-coded are APCs (green), naïve cells (light blue), pre-activated cells (dark blue), activated cells (orange), effector cells (red) and apoptotic cells (black). The diffusion field shows the IL-2 concentration in molars. (MOV 3448 kb) Additional file 6: CompuCell3D simulation of CD8^+^ T cells activation with weak antigenic stimulation plus 10 ng/ml IL-2 supplement. This movie is related to Fig. 3 in the main text and Additional file 2: Figure IVE. The color-coded are APCs (green), naïve cells (light blue), pre-activated cells (dark blue), activated cells (orange), effector cells (red) and apoptotic cells (black). The diffusion field shows the IL-2 concentration in molars. (MOV 8335 kb) Additional file 7: This zip file contains the simulation files used to generate the results presented in this manuscript. One may note that CompuCell3D must be installed in order to run the files. (ZIP 122 kb)
