# Supplementary material for: IL-2 sensitivity and exogenous IL-2 concentration gradient tune the productive contact duration of CD8+ T cell-APC: a multiscale modeling study
Source: BMC Syst Biol. 2016 Aug 17;10:77. doi: 10.1186/s12918-016-0323-y (PMC4989479; doi:10.1186/s12918-016-0323-y)
Supplement: Additional file 2: — This file contains the model implementation and parameterization. (DOCX 523 kb) [file 12918_2016_323_MOESM2_ESM.docx]

**Supplementary material**

**I. Model implementation and parameterization**

**Cellular level**

At cellular level, our model describes the behaviours of APCs and CD8^+^ T cells (Table I).

*Computational settings.* Simulations are performed in CompuCell3D simulation environment [2,3] on a square lattice. CompuCell3D implements a cellular Potts model, in which cells are represented as spatially-extended discrete objects, with explicit pixel-based shapes evolving throughout simulations. Cell interactions are characterized by an effective energy,

$$E= {\lambda_{perimeter}\sum_{\sigma>0} \left( p_{\sigma}- P_{\tau(\sigma)} \right)^{2}+ \lambda}_{area}\sum_{\sigma>0} \left( a_{\sigma}- A_{\tau(\sigma)} \right)^{2}+ \sum_{(x,y)} J_{\tau(\sigma\left( x \right)),\tau(\sigma(y))}\left( 1-\delta_{\sigma\left( x \right),\sigma(y)} \right)$$

where *σ* is an agent (a cell here), *τ*(*σ*) the type of agent *σ* (either an APC or one of the four different CD8^+^ T cell states considered, see below), and the effective energy of the system depends on perimeter constraints (first term in the formula), area constraints (second term), and interactions between neighbor pixels. Parameters *λ_perimeter_* and *λ_area_* define the strengths of perimeter and area constraints, respectively. Parameters *p_σ_* and *a_σ_* are the effective perimeter and area of agent *σ* while P*_τ_*_(_*_σ_*_)_ and A*_τ_*_(_*_σ_*_)_ are the expected perimeter and area for an agent of type *τ*(*σ*), respectively. The last term accounts for energy generated by interactions between neighbor pixels, with *J_τ_*_,_*_τ’_* the boundary energy per unit area between two agents of types *τ* and *τ’*, and *δ_σ_*_,_*_σ’_* equals 1 if and only if *σ* = *σ’* (that is, pixels belong to the same agent).

In order for cells to move, the effective energy of the system E is minimized at each simulation step (CompuCell3D uses Monte-Carlo steps). For a randomly chosen source node *x_s_*, a target node *x_t_* is selected in the vicinity of *x_s_* and the effective energy associated to the displacement from *x_s_* to *x_t_* is computed and compared to the current effective energy of the system. If the displacement minimizes the effective energy of the system, then the displacement is accepted; otherwise it is accepted with a probability depending on the energy cost.

*Space and time resolutions.* Simulations are performed on a *L*×*L* square lattice with periodic boundary conditions. Biological experiments show no evidence that mice lymph nodes are saturated with T cells (over the 72 hrs – 120 hrs pi period that we consider), hence the lattice size is set to *L*=200 pixels in order to avoid crowded environments (due for instance to proliferation). The initial size of a T cell is set to 4×4 pixels. Equalling the CD8^+^ T cell size 12 × 12 μm^2^ [4,5] yields a spatial resolution of 1 pixel = 3 × 3 μm^2^. In addition, the CD8^+^ T cell migration rate measured in the simulation is about 0.9 pixel length / simulation step, so using CD8^+^ T cell migration measurements *in vivo* (2-25 μm min^-1^ [6]) we set 1 minute ~ 3 Monte Carlo steps for the time scale.

*Initial conditions.* The initial time point of the simulations is 72 hrs pi, when CD8^+^ T cells are experimentally detectable in the draining lymph nodes. Due to computational cost, it is difficult to simulate the same number of cells than *in vivo*. Cell numbers have thus been normalized based on the cell count at the initial time point (i.e., 72 hrs pi). Naïve CD8^+^ T cells and APCs are initially randomly deployed in the computational domain for each simulation. Since it is difficult to measure the number of naïve CD8^+^ T cells in mice lymph nodes at the beginning of an immune response (72 hrs pi), we chose 30 CD8^+^ T cell and 3 APC as initial condition for each simulation, which was estimated from a study of *in vivo* imaging in draining lymph nodes [6] (CD8^+^ T cells:dendritic cells ratio of 10:1). Indeed, this T cell:APC ratio is appropriate to explain the average CD8^+^ T cell population dynamics *in vivo* (see Fig. 1B in the main text).

*Antigen-Presenting Cells (APC).* In biological experiments used to validate the model, mice received high dose (2.10^5^ TCID_50_/animal) of H1N1 Influenza virus. Hence, we assume that APC in the draining lymph nodes carry strong antigens that stimulate CD8^+^ T cells which then produce sufficient IL-2 for their activation, and that these responses are helper (CD4^+^) T cells independent [1]. In addition, we use the properties of dendritic cells to describe APC. Each APC has a larger cell size than T cells and migrates randomly with a slow speed in the domain (the quantitative properties of cells are detailed in Table III). APCs slowly move (2-3 μm min^-1^ [9]) and maintain a size of 250 pixels (corresponding to the average size of dendritic cells in collagen matrix 15-40 μm in length [5]). The frequency of APCs in lymph nodes is determined by several factors including the production of chemokines upon local inflammation, their life span and maturation duration [10]. For simplicity, we assume a constant number of APCs during day 3 - day 5 in the murine lymph node.

*CD8^+^ T cells.* Naïve CD8^+^ T cells initially in the computational domain (10 naïve cells for 1 APC, see above) are assumed to be antigen-specific cells, so they can all be activated by APC and non-antigen specific naïve cells are not described in this model. Activated and effector T cells, which are the only cell types that do proliferate in our model, are allowed unlimited proliferation provided they don’t die. The average cell cycle time of activated/effector CD8^+^ T cells was measured to be ~ 6 hours, and can reach as short as 2 hrs [7,8]. Therefore, we assign a random cycle time to each activated/effector CD8^+^ T cell by choosing from a uniform distribution *U*(2hrs, 10hrs).

**Subcellular level: molecular content partition at cell division**

At the end of division, the molecular content of the parental cell is split stochastically between daughter cells [11]. To this end, two daughter cells inherit the *k*_i_ and 2- *k*_i_ fraction of the mother cell molecule content. The exact range of partition ratio is unknown, we chose *k*_i_ $\in(0.7, 1.0)$for introducing a moderate stochasticity for all molecular components in Fig. I but T-bet. During the division of an activated CD8^+^ T cell, T-bet is asymmetrically partitioned between the two daughter cells [12]. To mimic such an asymmetric T-bet partition, *k*_i_ $\in(0.08, 0.2)$ is applied, based upon a quantitative measurement of fluorescence protein expression (cf. Fig. 1B in [12]).

Based on the initial level of T-bet in a dividing activated cell, it can either produce two effector cells (high T-bet initial levels), or one activated plus one effector cell (medium T-bet initial levels) or two activated cells (low T-bet initial levels).

We used *in vivo* measurements of the sum of Fas and its activated form Fas*, which is roughly constant (Fig. 1A), to predict the Fas* level (Fig. 1 in the main text) in our model by describing the transformation from Fas to Fas* (Eq. (4) in the main text).

**Extracellular level**

The description of the extracellular dynamics consists in the diffusion of IL-2. The diffusion coefficient for IL-2 (*D* in the equation of IL-2) was estimated at ~16 μm^2^ s^-1^ within a lymph node [13]. IL-2 has a serum half-life of 3.7 min +/- 0.8 min (mean +/- SD) in mice after i.v. injection [14], from which we estimated its decay constant δ is 0.187 min^-1^.

**Parameter estimation**

We calibrated parameter values by fitting the model to the data of CD8^+^ T cell population dynamics, mRNA profile *in vivo* ([www.ImmGen.org](http://www.ImmGen.org)) and the IL-2 concentration at single CD8^+^ T cell level. Starting from previous estimations of parameter values [15] as well as other parameter values, we varied parameter values individually in a fixed interval, centered on the initial value. For each tested parameter value, the residual sum of squares (RSS) was calculated for 10 simulations. Then the mean value of these 10 RSS values was computed and served as a fitting score. It must be noted that due to the stochastic nature of the model, small deviations of the parameter values do not significantly impair the fitting procedure (see examples in Section II dealing with sensitivity analysis). The procedure was repeated by modifying the length and central value of the intervals until the fitting score no longer improves, and parameter values minimizing the average value of the RSS were selected.

The main parameters and their values are summarized in Tables II, III and IV.

**Table I. Cell Behaviours.**

| **Behaviour**  **Cell type** | **Random motility** | **Replication** | **Apoptosis** |
| --- | --- | --- | --- |
| **APC** | **√** | **×** | **×** |
| **Naïve** | **√** | **×** | **×** |
| **Pre-activated** | **×** | **×** | **×** |
| **Activated** | **√** | **√** | **√** |
| **Effector** | **√** | **√** | **√** |

**Table II. Intracellular parameters (Key: M, molars).**

| **Parameter** | **Value** | **Definition** | **Reference** |
| --- | --- | --- | --- |
| **Decay rates:** | | | |
| *k*_R_ | 0.0023 min^-1^ | Decay rate of IL-2R | [1] |
| *k*_e_ | 0.0462 min^-1^ | Decay rate of IL-2•IL-2R | [1] |
| *k*_T_ | 0.0035 min^-1^ | Decay rate of T-bet | [16] |
| *k*_F_ | 0.0047 min^-1^ | Decay rate of Fas* | [17] |
| *k*_C_ | 0.0038 min^-1^ | Decay rate of Caspase | [17] |
|  |  |  |  |
| **Strength of feedbacks** | | | |
| *λ*_R1_ | 6.0 M min^-1^ | Strength of TCR inducing IL-2R expression | Derived |
| *λ*_R2_ | 0.5 min^-1^ | Self-induction strength of IL-2R expression | Derived |
| *λ*_T1_ | 0.36 M min^-1^ | Strength of TCR inducing T-bet expression | Derived |
| *λ*_T2_ | 0.018 min^-1^ | Self-induction strength of T-bet expression | Derived |
| *λ*_T3_ | 0.0035 M | Regulation constant of T-bet expression | Derived |
| *λ*_F_ | 3.47×10^-5^ M min^-1^ | Baseline rate of transcription of Fas* | [16] |
| *λ*_C1_ | 0.04 M min^-1^ | Strength of TCR inducting Caspase expression | Derived |
| *λ*_C2_ | 0.0005 M^-1^ | Strength of IL-2•IL-2R inhibiting Caspase expression | Derived |
| *λ*_C3_ | 0.001 | Strength of TCR inhibiting Caspase expression | Derived |
| *λ*_C4_ | 35 min^-1^ | Strength of Fas* inducing Caspase expression | Derived |
|  |  |  |  |
| **Association / Dissociation rates** | | | |
| $\mu_{IL2}^{+}$ | 6×10^8^ M^-1^ min^-1^ | Association rate of IL-2•IL-2R | [18] |
| $\mu_{IL2}^{-}$ | 0.0138 min^-1^ | Dissociation rate of IL-2•IL-2R | [19] |
| $H_{F}^{+}$ | 0.0002 M^-1^ min^-1^ | Activation rate of Fas | Derived |
| $H_{F}^{-}$ | 0.006 min^-1^ | Deactivation rate of Fas* | Derived |
|  |  |  |  |
| **Thresholds** | | | |
| IL-2•IL-2R | 51 M | Naïve cells become activated if crosses this threshold | Derived |
| T-bet | 3 M | Activated cells become effectors if cross this threshold | Derived |
| Caspase | 15 M | Cells become apoptosis if cross this threshold and | Derived |

**Table III. Cellular parameters.**

| **Phenotype** | **Diameter** | | **Migration speed** | | **Cycle time** | | **Life span** | |
| --- | --- | --- | --- | --- | --- | --- | --- | --- |
|  | **Value** | **Ref.** | **Value** | **Ref.** | **Value** | **Ref.** | **Value** | **Ref.** |
| APCs | 15-40 μm | [5] | 2-3 μm min^-1^ | [9] | / | / | 3 days | [20] |
| Naïve | 12 μm | [4,5] | 2-25 μm min^-1^ | [6] | / | / | >232 days | [21] |
| Pre-Activated | 12 μm | [4,5] | / | / | / | / | / | / |
| Activated | 12 μm | [4,5] | 2-25 μm min^-1^ | [6] | 2-10 hrs | [7,8] | / | / |
| Effector | 12 μm | [4,5] | 2-25 μm min^-1^ | [6] | 2-10 hrs | [7,8] | / | / |

**Table IV. Extracellular parameters (Key: M, molars)**

| **Parameter** | **Value** | **Definition** | **Reference** |
| --- | --- | --- | --- |
| **Equation for IL-2** |  |  |  |
| *D* | 960 μm^2^ min^−1^ | Diffusion coefficient of IL-2 in serum | [13] |
| *δ* | 0.187 min^−1^ | Decay rate of IL-2 in serum | [14] |
| *λ*_1_ | 2×10^-10^ M min^-1^ | Strength of TCR inducing IL-2 production | [13] |
| *λ*_R3_ | 10^-5^ M min^-1^ | Strength of IL-2•IL-2R inducing IL-2 production | Derived |
| *λ*_R4_ | 10^-5^ M | Strength of IL-2•IL-2R inhibiting IL-2 production | Derived |
| *λ*_T4_ | 10^-5^ M^-1^ | Strength of T-bet inhibiting IL-2 production | Derived |

**II. Sensitivity analysis for thresholds of CD8^+^ T cell activation, effector formation and apoptosis**

By changing the threshold value in a fix interval, we analysed the subsequent changes in terms of total cell number, percentage of CD8^+^ effector T cells and protein expressions. In Fig. I, II and III, root mean squared deviation (RMSD) is calculated to measure the distance between simulation results and the *in vivo* mRNA data at 72 hrs, 96 hrs and 120 hrs pi.

**IL-2•IL-2R threshold**

The change of IL-2•IL-2R threshold level can modulate cell population size (Fig. IA) through the control of the pre-activation/activation transitional checkpoint. However, the IL-2•IL-2R threshold changes have no significant impact on the fraction of effector cells in the cell population (Fig. IB). Since the IL-2•IL-2R threshold changes affect the T cell-APC contact duration (Fig. 7A in the main text), it influences the kinetics of TCR-dependent signalling pathways (e.g., T-bet) (Fig. IC) which consequently affects the population dynamics. For example, a low IL-2•IL-2R threshold limits the T-bet expression by shortening the T cell activation time; the reduced T-bet expression (comparing to the control level) decreases FasL expression which limits the Caspase activity; in consequence, a resulted low death rate contributes to a large size population (Fig. IA).

**T-bet threshold**

T-bet threshold changes have no significant impact on the total cell number (Fig. IIA). A higher T-bet threshold leads to a decrease in the percentage of effector cells (Fig. IIB) since it reduces the chance of effector cell development. T-bet threshold change has no significant effect on intracellular dynamics (Fig. IIC).

**Caspase threshold**

A lower Caspase threshold level results in quick cell death and eventually a smaller population size (Fig. IIIA). Caspase threshold changes do not affect the fraction of effector cells (Fig. IIIB). A lower Caspase threshold leads to a decreased level of intracellular signals (Fig. IIIC), because rapid cell apoptosis leaves short time for molecule expression.

**Fig. I***.* Sensitivity analysis of IL-2•IL-2R threshold in terms of **(A)** total population size (mean±SD; n=10 simulations), **(B)** percentage of effectors (mean±SD; n=10 simulations) and **(C)** intracellular dynamics (mean±SD; n=10 simulations). (Key: ‘C’ in x-axis means control values corresponding to Fig. 3 in the main text.)

**Fig. II***.* Sensitivity analysis of T-bet threshold in terms of **(A)** total population size (mean±SD; n=10 simulations), **(B)** percentage of effectors (mean±SD; n=10 simulations) and **(C)** intracellular dynamics (mean±SD; n=10 simulations). (Key: ‘C’ in x-axis, control values corresponding to Fig. 3 in the main text.)

**Fig. III***.* Sensitivity analysis of Caspase threshold in terms of **(A)** total population size (mean±SD; n=10 simulations), **(B)** percentage of effectors (mean±SD; n=10 simulations) and **(C)** intracellular dynamics (mean±SD; n=10 simulations). (Key: ‘C’ in x-axis, control values corresponding to Fig. 3 in the main text.)

**III. Intracellular dynamics in different TCR-strength and IL-2 concentration stimulatory conditions.**

**
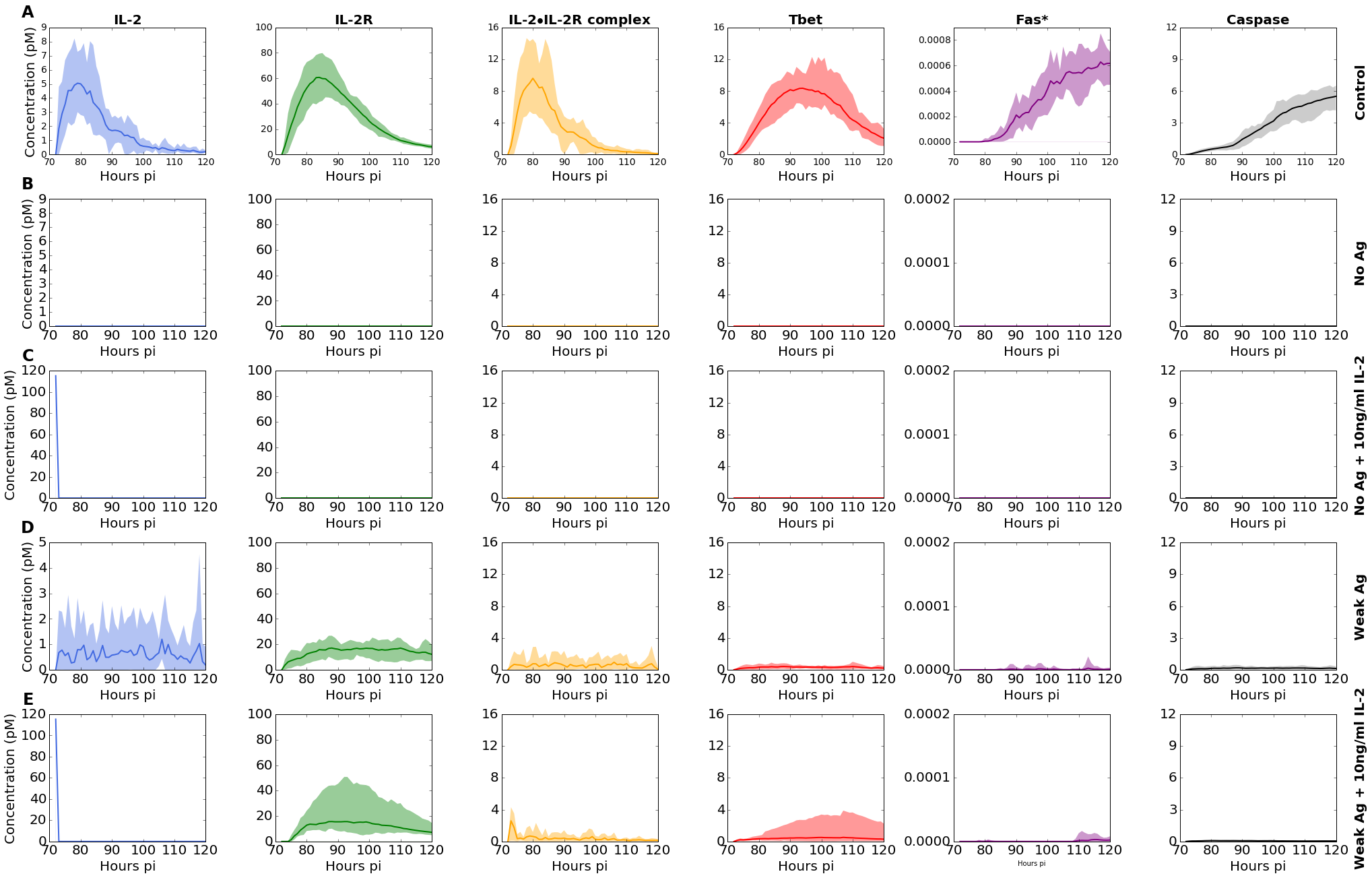
**

**Fig. IV**. Intracellular dynamics of CD8^+^ T cells with **(A)** strong antigenic stimulation, **(B)** antigen-free condition, **(C)** antigen-free condition plus 10 ng/ml IL-2, **(D)** weak antigenic stimulation and **(E)** weak antigenic stimulation plus 10 ng/ml IL-2.

**IV. References**

1. Duprez V, Cornet V, Dautry-Varsat A. Down-regulation of high affinity interleukin 2 receptors in a human tumor T cell line. Interleukin 2 increases the rate of surface receptor decay. J Biol Chem. 1988;263: 12860–12865. pmid: 3138231.

2. Swat MH, Hester SD, Balter AI, Heiland RW, Zaitlen BL, Glazier JA. Multi-Cell Simulations of Development and Disease Using the CompuCell3D Simulation Environment. Methods Mol Biol. 2009;500: 361–428. doi: 10.1007/978-1-59745-525-1_13. pmid: 19399437.

3. Swat MH, Thomas GL, Belmonte JM, Shirinifard A, Hmeljak D, Glazier JA. Multi-scale modeling of tissues using CompuCell3D. Methods Cell Biol. 2012;110: 325–366. doi: 10.1016/B978-0-12-388403-9.00013-8. pmid: 22482955.

4. Negulescu PA, Krasieva TB, Khan A, Kerschbaum HH, Cahalan MD. Polarity of T cell shape, motility, and sensitivity to antigen. Immunity. 1996;4: 421–430. doi: 10.1016/S1074-7613(00)80409-4. pmid: 8630728.

5. Friedl P, Zänker KS, Bröcker EB. Cell migration strategies in 3-D extracellular matrix: differences in morphology, cell matrix interactions, and integrin function. Microsc Res Tech. 1998;43: 369–378. doi: 10.1002/(SICI)1097-0029(19981201)43:5<369::AID-JEMT3>3.0.CO;2-6. pmid: 9858334.

6. Miller MJ, Wei SH, Cahalan MD, Parker I. Autonomous T cell trafficking examined in vivo with intravital two-photon microscopy. Proc Natl Acad Sci USA. 2003;100: 2604–2609. doi: 10.1073/pnas.2628040100. pmid: 12601158.

7. Kaech SM, Wherry EJ, Ahmed R. Effector and memory T-cell differentiation: implications for vaccine development. Nat Rev Immunol. 2002;2: 251–262. doi:10.1038/nri778. pmid: 12001996.

8. Yoon H, Kim TS, Braciale TJ. The Cell Cycle Time of CD8+ T Cells Responding In Vivo Is Controlled by the Type of Antigenic Stimulus. PLoS ONE. 2010;5: e15423. doi: 10.1371/journal.pone.0015423. pmid: 21079741.

9. Lindquist RL, Shakhar G, Dudziak D, Wardemann H, Eisenreich T, Dustin ML, et al. Visualizing dendritic cell networks in vivo. Nat Immunol. 2004;5: 1243–1250. doi: 10.1038/ni1139. pmid: 15543150.

10. Banchereau J, Briere F, Caux C, Davoust J, Lebecque S, Liu Y-J, et al. Immunobiology of Dendritic Cells. Annu Rev Immunol. 2000;18: 767–811. doi:10.1146/annurev.immunol.18.1.767. pmid: 10837075.

11. Warren G. Membrane partitioning during cell division. Annu Rev Biochem. 1993;62: 323–348. doi: 10.1146/annurev.bi.62.070193.001543. pmid: 8352593.

12. Chang JT, Ciocca ML, Kinjyo I, Palanivel VR, McClurkin CE, Dejong CS, et al. Asymmetric proteasome segregation as a mechanism for unequal partitioning of the transcription factor T-bet during T lymphocyte division. Immunity. 2011;34: 492–504. doi: 10.1016/j.immuni.2011.03.017. pmid: 21497118.

13. Höfer T, Krichevsky O, Altan-Bonnet G. Competition for IL-2 between Regulatory and Effector T Cells to Chisel Immune Responses. Front Immunol. 2012;3: 268. doi: 10.3389/fimmu.2012.00268. pmid: 22973270.

14. Donohue JH, Rosenberg SA. The fate of interleukin-2 after in vivo administration. J Immunol. 1983;130: 2203–2208. pmid: 6601147.

15. Prokopiou S, Barbarroux L, Bernard S, Mafille J, Leverrier Y, Arpin C, et al. Multiscale Modeling of the Early CD8 T-Cell Immune Response in Lymph Nodes: An Integrative Study. Computation. 2014;2(4): 159–181. doi: 10.3390/computation2040159.

16. Yates A, Callard R, Stark J. Combining cytokine signalling with T-bet and GATA-3 regulation in Th1 and Th2 differentiation: a model for cellular decision-making. J Theor Biol. 2004;231: 181–196. doi: 10.1016/j.jtbi.2004.06.013. pmid: 15380383.

17. Larsson E, Sander C, Marks D. mRNA turnover rate limits siRNA and microRNA efficacy. Mol Syst Biol. 2010;6: 433. doi: 10.1038/msb.2010.89. pmid: 21081925.

18. Wang HM, Smith KA. The interleukin 2 receptor. Functional consequences of its bimolecular structure. J Exp Med. 1987;166: 1055–1069. doi: 10.1084/jem.166.4.1055. pmid: 3116143.

19. Feinerman O, Jentsch G, Tkach KE, Coward JW, Hathorn MM, Sneddon MW, et al. Single-cell quantification of IL-2 response by effector and regulatory T cells reveals critical plasticity in immune response. Mol Syst Biol. 2010;6: 437. doi: 10.1038/msb.2010.90. pmid: 21119631.

20. Hou W-S, Van Parijs L. A Bcl-2-dependent molecular timer regulates the lifespan and immunogenicity of dendritic cells. Nat Immunol. 2004;5: 583–589. doi: 10.1038/ni1071. pmid: 15133508.

21. McCune JM, Hanley MB, Cesar D, Halvorsen R, Hoh R, Schmidt D, et al. Factors influencing T-cell turnover in HIV-1-seropositive patients. J Clin Invest. 2000;105: R1–8. doi: 10.1172/JCI8647. pmid: 10712441.
